# MiR-302a Limits Vascular Inflammation by Suppressing Nuclear Factor-κ B Pathway in Endothelial Cells

**DOI:** 10.3389/fcell.2021.682574

**Published:** 2021-08-02

**Authors:** Jia-Ni Yuan, Yu Hong, Zhuo-Lin Ma, Rui-Ping Pang, Qing-Qing Lei, Xiao-Fei Lv, Jia-Guo Zhou, Hui Huang, Ting-Ting Zhang

**Affiliations:** ^1^Program of Cardiovascular Research, The Eighth Affiliated Hospital, Zhongshan School Medicine, Sun Yat-sen University, Guangzhou, China; ^2^Department of Pharmacology, and Cardiac and Cerebral Vascular Research Center, Zhongshan School of Medicine, Sun Yat-sen University, Guangzhou, China; ^3^Department of Physiology, Zhongshan School Medicine, Sun Yat-sen University, Guangzhou, China; ^4^Department of Cardiology, The Eighth Affiliated Hospital, Sun Yat-sen University, Shenzhen, China

**Keywords:** MicroRNA-302a, vascular inflammation, endothelial cells, NF-κB, IRAK4, ZFP91

## Abstract

The inflammatory response of endothelial cells accelerates various vascular diseases. MicroRNAs (miRNAs) participate in diverse cellular processes during inflammation. In the present study, we found that miR-302a is an effective suppressor of vascular inflammation in endothelial cells. It was revealed that miR-302a exhibited a lower level in a lipopolysaccharide (LPS)-induced mouse model and in patients with vascular inflammatory disease. Genetic haploinsufficiency of miR-302 aggravated the LPS-induced vascular inflammatory response in mice, and overexpression of miR-302a attenuated vascular inflammation in mice. Furthermore, overexpression of miR-302a inhibited the synthesis and secretion of adhesion factors in endothelial cells, and suppressed the adhesion of monocytes to endothelium. In the study of molecular mechanism, we found that miR-302a relieved vascular inflammation mainly by regulating the nuclear factor kappa-B (NF-κB) pathway in endothelial cells. The results showed that interleukin-1 receptor-associated kinase4 (IRAK4) and zinc finger protein 91 (ZFP91) were the binding targets of miR-302a. MiR-302a prevented the nuclear translocation of NF-κB by inhibiting phosphorylation of IκB kinase complex β (IKKβ) and inhibitors of κBα (IκBα) via targeting IRAK4. In addition, miR-302a downregulated the expression of NF-κB by directly binding with ZFP91. These findings indicate that miR-302a negatively regulates inflammatory responses in the endothelium via the NF-κB pathway and it may be a novel target for relieving vascular inflammation.

## Introduction

Inflammation contributes to various vascular diseases such as coronary heart disease, atherosclerosis, arteritis, and aneurysms (Paulus and Tschöpe, 2013; Maegdefessel et al., 2014; O’Neill et al., 2015; Bäck et al., 2019). Abnormal activation of endothelial cells can accelerate vascular inflammation (Yang et al., 2016). Activated endothelial cells secrete adhesion factors and release more inflammatory factors into serum, which recruit inflammatory cells to the injury sites. The accumulation of inflammatory cells and increased inflammatory factors aggravate vascular inflammation and ultimately induce inflammatory damage in other organs such as the liver and lungs (Yang et al., 2012). Therefore, inhibiting inflammation in endothelial cells is an important strategy in preventing vascular inflammatory diseases.

MicroRNAs (miRNAs) are a family of endogenous noncoding small RNAs consisting of 18–24 nucleotides which regulate gene expression at the post-transcriptional level (Saliminejad et al., 2019). MiR-302 was initially identified in human embryonic stem cells and plays a vital role in these cells, and mice deficient in miR-302 have a fully penetrant late embryonic lethal phenotype (Barroso-delJesus et al., 2008). MiR-302a belongs to the miR-302 family which is encoded by the miR-302–367 cluster and includes miR-302a, miR-302b, miR-302c, miR-302d, miR-302e, and miR-367 (Barroso-del Jesus et al., 2009). Studies have identified the function of miR-302a in embryonic development and tumor invasion (Chen et al., 2019; Jiang et al., 2020; Ma et al., 2018; Yu et al., 2020). However, the role of miR-302a in cardiovascular disease has rarely been reported. Our previous studies showed that miR-302a exacerbates the proliferation and restenosis of smooth muscle cells (Liu et al., 2020), but the function of miR-302a in endothelial cells remains unclear.

Nuclear factor kappa-B (NF-κB) is a nuclear transcription factor mainly involved in the processes of inflammation and immunity. A study showed that miR-302e can prevent allergic inflammation by inhibiting the activation of NF-κB in a human mast cell line. Additionally, miR-302a-3p, a mature sequence of miR-302a, can inhibit receptor activator of NF-κB ligand which regulates the activation of NF-κB by binding with it (Irwandi et al., 2018); thus, miR-302a might be an essential regulator of the NF-κB pathway. On the basis of the above research, we hypothesized that miR-302a regulates endothelial inflammation via the NF-κB pathway. In the present study, we found that miR-302a suppressed the inflammatory response in the endothelium and inhibited activation of the NF-κB pathway through ZFP91 and IRAK4-IKKβ-IκBα signaling.

## Materials and Methods

### Ethics Statement

The study protocol was approved by the Medical Research Ethics Committee of Sun Yat-sen University. Animal experiment strictly followed the principle to minimize the pain, suffering, and discomfort to experimental animals.

### Reagents and Antibodies

HDMEM high glucose medium, M199 medium, 1,640 medium were purchased from Gibco. The human mononuclear macrophage cell line THP-1 was purchased from the cell bank of the Type Culture Collection Committee of the Chinese Academy of Sciences. Human TNF-α and LPS were purchased from Sigma. Calcein-AM was purchased from Invitrogen. The human umbilical cord was taken from the delivery room of the First Affiliated Hospital of Sun Yat-sen University. ICAM-1, ZFP91, β-actin, GAPDH, and Ly-6G antibodies were purchased from Abcam. IRAK4, NF-κB p65, *p*-IκBα, IκBα, *p*-IKKβ, IKKβ, VCAM-1, CD3, and F4/80 antibodies were all purchased from Cell signaling technology company. Lamin B antibody was purchased from Santa cruz company. ELISA kit to evaluate ICAM-1, VCAM-1, IL-6, MCP-1, IFN-γ, and E-selectin were from Boster Biological Engineering Co (Wuhan, China). Adenovirus expression of negative control or miR-302a-mimics were from Genepharma Co (Shanghai, China). NE-PER^®^ nuclear and cytoplasmic extraction reagents for nuclear and cytoplasmic proteins extraction was from Thermo Scientific (Waltham, MA, United States).

### Human Plasma Samples

Human plasma samples were obtained from patients with coronary artery disease (*n* = 9), and healthy controls (*n* = 9). Patients with related cardiovascular diseases were excluded from the group. This study was approved by the Medical Research Ethics Committee of Sun Yat-sen University. Informed consent was obtained from all subjects and the experiments were conducted according to the principles expressed in the Declaration of Helsinki.

### MiR-302 Heterozygous Mice

MiR-302 heterozygous mice (miR-302^+/–^) were generated on the C57BL/6 background at Nanjing Model Animal Research Center as previously described (Liu et al., 2020). Briefly, CRISPR/Cas9 gene targeting method was used for construction of MiR-302 heterozygous mice (miR-302^+/–^) on the C57BL/6 background at Nanjing Model Animal Research Center. sgRNA mRNA and linearized Cas9-encoding vector were microinjected into the cytoplasm of the zygote, which was transferred into the oviductal ampullae of female mice. Tail DNA of mice were extracted for genotyping by PCR using specific primers as follows. The first pair of primers: Mir302a-s-loxp-tF2:5′ttattgactgggcttcccaccac3′, Mir302a-s-loxp-tR2: 5′actgacacaggtccatcaccattg3′; The second pair of primers: Neo-3F:5′tctgaggcggaaagaaccag3′, Mir302a-s-loxp-tR2: 5′actgacacaggtccatcaccattg3′.

### Animal Experiments

To investigate the role of miR-302a in inflammation, 8-weeks wild-type mice (WT), miR-302^+/–^ mice and WT mice injected with adenovirus expression of negative control or miR-302a-mimics were measured in basal condition. In LPS-induced inflammatory mice model, 8 weeks old mice (WT mice, miR-302^+/–^ mice, WT mice injected with adenovirus expression of negative control or miR-302a-mimics) were divided into groups randomly, the mice were intraperitoneally injected with 25 mg/kg LPS, after 96 h the mice were sacrificed with pentobarbital sodium, the tissues and serum were then collected. All animals were kept in the SPF animal laboratory of the Experimental Animal Center of Sun Yat-sen University North Campus. This experiment was approved by the Animal Ethics Committee of Sun Yat-sen University.

### Transfection of Plasmids

MiR-302a-mimics/inhibitor and negative control (mimics/inhibitor negative control) powders were diluted into a final concentration of 20 μM with RNase-free DEPC water and then stored at −20°C. The umbilical vein endothelial cells were seeded in 35 mm dishes at a density of 2 × 10^5^ cells/ml. They were washed three times with PBS before transfection. Every dish was added with 700 μl serum-free medium, 6 μl hiperfect transfection reagent and mimics/inhibitor of different concentration. Mimics/inhibitor were mixed in 100 μl serum-free medium for 10 min at room temperature, then the mixture was dropped into the small dish slowly and cultured in a 5% CO_2_, 37°C incubator. All dishes were supplemented with 1.2 ml of complete medium after 3 h and extracted after 48 h. MiR-302a-mimics/inhibitor are synthesized by Jima Pharmaceutical Technology Co., Ltd (Shanghai, China). The plasmids of psiCHECK-2-p65, ZFP91 3′UTR, and IRAK4 3′UTR were constructed by Heyuan Biotechnology Co. Ltd (Shanghai, China). Lipofectamine 2000 transfection reagent was purchased from Invitrogen.

### Isolation and Culture of Human Umbilical Vein Cells

Healthy baby umbilical cord about 20 cm was soaked in sterile PBS within 12 h after parturition. Next, a large gavage needle was inserted to the umbilical vein hole and hemostatic forceps were used to clamp it. Then, the blood vessel was rinsed three times until rinsing the residual blood flows all out with sterile PBS and 50 ml syringe. After that, the outer wall of the umbilical cord was wiped with an alcohol cotton ball, while the other end of the umbilical cord was clamped with a hemostatic forceps. The digestive fluid containing 0.125% pancreatin and 0.01% EDTA was injected into the blood vessel with 50 ml syringe through the gavage needle. The blood vessel was digested in 37°C incubator for about 7 min, then the digestion solution was collected into a beaker containing a small amount of complete medium (M199 contains 20% FBS, heparin 2,500 U, 100 U/ml penicillin, 100 U/ml streptomycin, 75 μg/ml FGF, and 2 mM glutamine). Blood vessels were washed twice with PBS to promote the shedding of endothelial cells, and the lavage fluid was collected in a beaker. Then, the digestive and lavage fluid were mixed, poured into 15 ml centrifuge tubes, and centrifuged at 1,000 r/min for 5 min. The supernatant was discarded, and the cells were resuspended with an appropriate amount of complete medium. At last, the cells were planted in a cell culture flask, cultured in a 5% CO_2_, 37°C incubator. After 6 h, the cells were replaced with a new medium to remove non-adherent cells and residual blood cells. After that, the culture medium was changed every 2 days, and the cells were visible in short spindle or cobblestone-like mosaic arrangement after about 5–7 days. The human umbilical cord was taken from the delivery room of the First Affiliated Hospital of Sun Yat-sen University.

### MiRNA Target Prediction

Base-pairing for the binding of the candidate miRNA to the targeted gene sequences was predicted using the TargetScan v.7.0^1^.

### Adhesion Assay of Monocytes and Endothelial Cells

Human umbilical vein cells (HUVECs) were planted in a confocal dish at a density of 2 × 10^5^ cells/ml as they filled up 90% of the entire flask. THP-1 cells were loaded with Calcein-AM green fluorescent probe in a final concentration of 3 μm/L, incubated at 37°C in the dark for 30 min, and then resuspended in RPMI-1640 medium after washed twice with PBS. In the adhesion assay, THP-1 cells labeled with green fluorescence and HUVECs were incubated at 37°C incubator in the dark, cells were washed gently twice with PBS to wash away unadhered monocytes after 1 h. Finally, 1 ml of 1,640 culture medium was added to each dish and observed under a laser confocal microscope at an excitation wave length of 485 nm and an emission wavelength of 530 nm.

### Quantitative Real-Time PCR

Total RNA was extracted from cells or tissues with Trizol reagent (Invitrogen, United States). extracted RNA was reversed into cDNA with High Capacity cDNA Reverse Transcription Kit (Applied Biosystems, United States). Real-time PCR was performed using SYBR Green fluorescence (ABI, United States) as previously described (Su et al., 2019). After amplification, the threshold cycle (Ct) was determined. Primer sequence for experiments: (1) IRAK4 Primer F: 5′gtcatgaccagccgaatcgtg3′. Primer R: 5′cagacactggtcagcagcaga3′; (2) p65 Primer F: 5′ggtccacggcggaccggt3′. Primer R: 5′gaccccgagaacgtggtgcgc3′; (3) ZFP91 Primer F: 5′tgcgacatgcgaaacatcata3′. Primer R: 5′gtgtactgccagattgtgggaac3′; (4) miR-302a Primer F: 5′aataagtgcttccatgttttggtga3′; and U6 forward: 5′attggaacgatacagag3′, reverse: 5′ggaacgcttcacgaatttg3′. The fold change in gene expression was calculated using the 2^–ΔΔCT^ method, with 18S rRNA or U6 as an internal control.

### Examination of Luciferase Activity

The luciferase activity was detected according to the method provided by Promega’s dual luciferase detection kit. After 48 h of transfection, the cells were washed twice with PBS, and 100 μl of PLB (Passive Lysis Buffer) was added to each well. Next, each well was shaken gently at room temperature for 15 min, and cell lysate was collected. After adding 20 μl of cell lysate to the luminescent plate, read the background value with a GloMax bioluminescence detector for 2 s; add 100 μl of LAR II working solution to each sample, mix quickly, and read the value for 2 s; then, add 100 μl Stop and Glo to each sample^®^ Reagent, after mixing quickly, put it into the luminescence detector and read the value for 2 s. The dual luciferase reporter gene detection kit was purchased from Promega.

### Statistical Analysis

The data in all experiments were in accordance with the normal distribution, values are presented as mean ± SEM. Since the experimental data were mostly pairwise comparisons between groups and met the requirements of normal distribution and homogeneity of variance, *t*-test or one-way statistical analysis of variance was used. *p* < 0.05 was considered statistically different.

## Results

### MiR-302a Was Decreased in the Inflammatory Response

**FIGURE 1:**
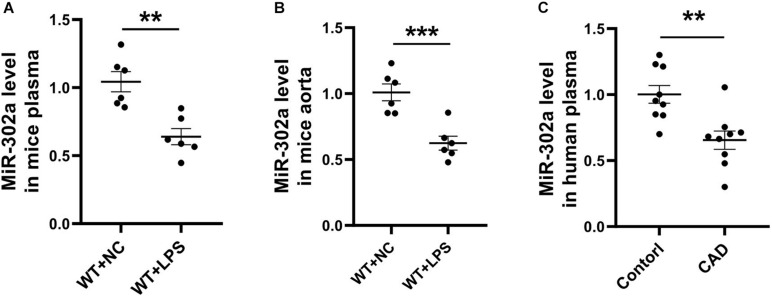
MiR-302a is decreased in serum and vascular tissue under inflammatory condition. wild-type (WT) mice were intraperitoneally injected with 25 mg/kg LPS, mRNA level of miR-302a in serum **(A)** and aorta **(B)** of mice was detected by quantitative PCR after 96 h [**p < 0.01; ***p < 0.001 vs. Negative Control (WT + NC) *n* = 6]. **(C)** MiR-302a level in coronary heart disease (CAD) patients and control donors (Control) was examined by quantitative PCR (**p < 0.01 vs. control *n* = 9).

To determine the role of miR-302a in vascular inflammation, lipopolysaccharide (LPS), which promotes the synthesis of cytokines and has long been used for classical inflammatory model induction (Wang L. et al., 2017; Wang X. et al., 2017), was injected into mice in order to construct an inflammation model. After 96 h, mouse serum and aorta tissue were obtained and analyzed. Quantitative PCR (qPCR) results suggested that miR-302a was decreased under LPS-induced inflammation (Figures 1A,B). Moreover, considering that coronary heart disease (CAD) is a representative vascular inflammatory disease and is mainly caused by endothelial damage (Gimbrone and García-Cardeña, 2016), we collected the serum of CAD patients and healthy donors. The qPCR data showed that miR-302a was also markedly reduced in CAD patients (Figure 1C). These data suggested that miR-302a exhibited a lower level under the vascular inflammatory state in humans and mice, indicating its possible key role in regulating inflammation.

### MiR-302 Deficiency Aggravated the LPS-Induced Vascular Inflammatory Response

**FIGURE 2:**
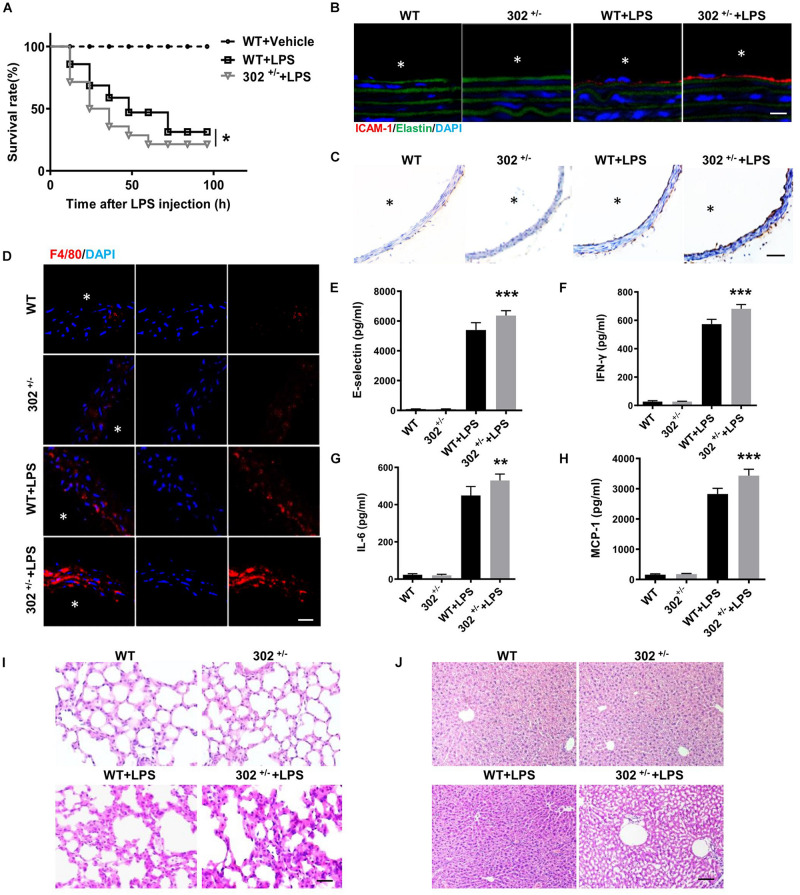
Deficiency of miR-302 accelerates lipopolysaccharide (LPS)-induced vascular inflammation. **(A)** The survival rate of 8-weeks miR-302^+/−^ mice (302^+/−^) and WT mice after LPS injection (25 mg/kg), *n* = 20. **(B)** The expression of ICAM-1 in aorta from WT and miR-302^+/−^ mice was detected by immunofluorescence, *n* = 5, scale bar, 10 μm, asterisk indicates the lumen of aorta. **(C)** Immunohistochemical staining of aorta VCAM-1 from WT and miR-302^+/−^ mice injected with LPS, *n* = 6, scale bar, 100 μm. **(D)** Immunofluorescence staining of F4/80 represents the infiltration of macrophages (red) in the aorta, nuclei were counterstained with DAPI (blue), *n* = 5, scale bar, 50 μm. The level of inflammatory factors E-selectin **(E)**, IFN-γ **(F)**, IL-6 **(G)**, and MCP-1 **(H)** in serum from miR-302^+/−^ mice and WT mice, *n* = 6. The inflammatory status of lung **(I)** and liver **(J)** stained by HE staining, *n* = 6. Scale bar, 100 μm (*p < 0.05; **p < 0.01; ***p < 0.001 vs. WT + LPS).

To determine whether miR-302a is an initial regulator of inflammation, we constructed constitutive miR-302a deficient mice, and as homozygous miR-302 knockout mice were unavailable, heterozygous mice (miR-302^+/–^) were used in the study (Supplementary Figure 1). When the mice were injected intraperitoneally with LPS, HE staining showed that miR-302a deficiency had no obvious effect on vascular structure (Supplementary Figure 2). But the survival rate of miR-302^+/–^ mice was significantly lower than that of WT mice after 96 h (Figure 2A). More importantly, LPS-induced expression of adhesion factors including vascular cell adhesion molecule 1 (VCAM-1) and intercellular adhesion molecule 1 (ICAM-1) in the aorta were significantly upregulated in miR-302^+/–^ mice compared with WT group (Figures 2B,C). Meanwhile, more F4/80-positive macrophages infiltrated in the aorta of miR-302^+/–^ mice (Figure 2D). These data demonstrated that deficiency of miR-302 aggravated LPS-induced inflammation in the aorta.

It has been reported that upregulation of inflammatory cytokines, such as interleukin (IL)-6, monocyte chemoattractant protein (MCP)-1, interferon (IFN)-γ, and E-selectin, are markers of inflammation and endothelial dysfunction, and the plasma levels of these cytokines are frequently increased in various cardiovascular diseases including CAD (Dame et al., 2007; von Scholten et al., 2016; Rahman et al., 2017; Zhou et al., 2017). Using ELISA kits, we found that the levels of these cytokines in serum were markedly increased in miR-302^+/–^ mice compared with WT (Figures 2E–H). Consistently, LPS-induced inflammatory response in the liver and lung was also more obvious in miR-302^+/–^ mice than that in WT mice (Figures 2I,J). Our data showed that the infiltration of Ly-6g positive neutrophils, F4/80 positive macrophages and CD3 positive T cells, as well as the activity of myeloperoxidase (MPO) (Supplementary Figure 3), a specific marker of myeloid cells (Greenhalgh et al., 2018), were all significantly increased in the liver and lung of miR-302^+/–^ mice compared with WT group. These findings demonstrated that miR-302 deficiency aggravated LPS-induced inflammatory response.

### Overexpression of MiR-302a Attenuated the LPS-Induced Vascular Inflammatory Reaction

**FIGURE 3:**
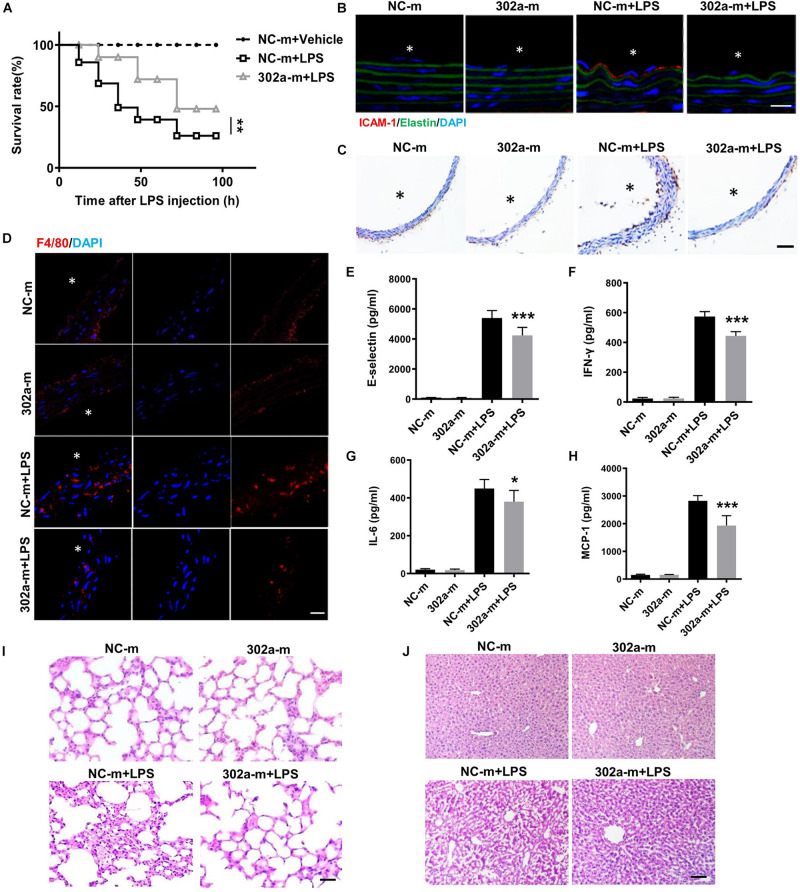
MiR-302a-mimics weakens LPS-induced inflammation of mice. **(A)** The survival rate of 8 weeks’ WT mice injected with miR-302a mimics (302a-m) or negative control mimics (NC-m). WT mice were injected with LPS (25 mg/kg), *n* = 20. **(B)** Immunofluorescence of ICAM-1 in aorta from mice injected with miR-302a mimics or negative control mimics. *n* = 6, scale bar, 10 μm, asterisk indicated the lumen of aorta. **(C)** Immunohistochemical staining of VCAM-1 of aorta from mice injected with mimics. *n* = 5, scale bar, 100 μm. **(D)** Immunofluorescence staining of F4/80 represents the infiltration of macrophages (red) in the aorta, nuclei were counterstained with DAPI (blue), *n* = 6, scale bar, 50 μm. The inflammatory factors of E-selectin **(E)**, IFN-γ **(F)**, IL-6 **(G)**, and MCP-1 **(H)** in serum of mice (*n* = 6). The inflammatory status in lung **(I)** and liver **(J)** stained by HE, *n* = 6, scale bar, 100 μm (*p < 0.05; **p < 0.01; ***p < 0.001 vs. NC-m + LPS).

To further examine whether upregulating miR-302a can inhibit the inflammatory response, we proceeded systemic delivery of miR-302a-mimics in mice by tail vein injection. The results suggested that miR-302a in aorta intima was evidently overexpressed in mice injected with miR-302a-mimics comparing to control group (Supplementary Figure 4). Similarly, in LPS-induced inflammatory condition, HE staining showed that miR-302a mimics had no obvious effect on vascular structure (Supplementary Figure 5), but miR-302a overexpression reduced the mortality of mice significantly (Figure 3A). Immunostaining results suggested that miR-302a mimics decreased the LPS-induced expression and secretion of ICAM-1 and VCAM-1 especially in aortic endothelium (Figure 3B,C). Additionally, overexpressing miR-302a inhibited the infiltration of F4/80-positive macrophages evidently in mice aorta (Figure 3D). The inflammatory factors E-selectin, IL-6, MCP-1, and IFN-γ were also downregulated by miR-302a (Figures 3E–H). Consistently, LPS-induced inflammatory response in the liver and lung was alleviated in mice injected with miR-302a-mimics comparing to control group (Figures 3I,J). Moreover, the infiltration of Ly-6g positive neutrophils, F4/80 positive macrophages and CD3 positive T cells, as well as the activity of MPO were all significantly decreased in the liver and lung by upregulation of miR-302a (Supplementary Figure 6). These results revealed that miR-302a is effective in suppressing the LPS-induced vascular inflammatory response.

### MiR-302a Inhibited the Secretion of Adhesion Factors and Adhesion of Monocytes With Endothelial Cells

**FIGURE 4:**
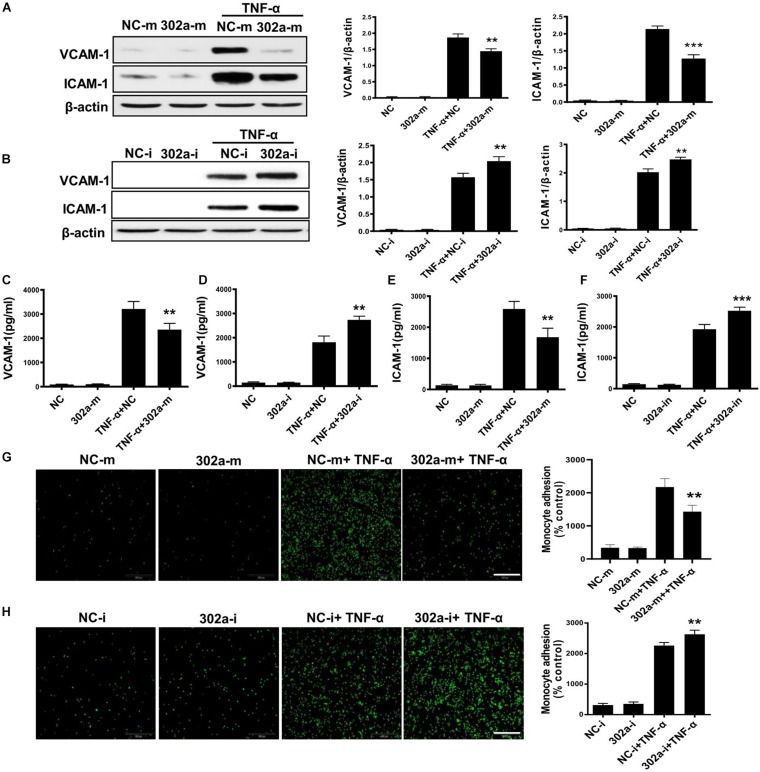
MiR-302a inhibits secretion of adhesion factors and adhesion of monocytes to endothelial cells. **(A)** VCAM-1 and ICAM-1 protein levels in Human Umbilical Vein Cells (HUVECs) transfected with 25 nM miR-302a-mimics/NC-mimics after TNF-α (10 ng/ml) treatment for 12 h, *n* = 5. **(B)** VCAM-1 and ICAM-1 were detected by western blot in HUVECs transfected with 100 nM miR-302a-inhibitor/NC-inhibitor after TNF-α (10 ng/ml) treatment for 12 h. *p < 0.05; **p < 0.01; ***p < 0.001 vs. TNF-α + NC, *n* = 5. The secretion of VCAM-1 **(C)** and ICAM-1 **(D)** in HUVECs culture medium detected by ELISA after transfected with miR-302a-mimics/NC-mimics (25 nM) for 36 h and then treated with 10 ng/ml TNF-α for 12 h. *p < 0.05; **p < 0.01; ***p < 0.001 vs. TNF-α + NC, *n* = 5. The expression of VCAM-1 **(E)** and ICAM-1 **(F)** in HUVECs culture medium detected by ELISA after transfected with miR-302a-inhibitor/NC-inhibitor (100 nM) for 36 h and then treated with 10 ng/ml TNF-α for 12 h. *p < 0.05; **p < 0.01; ***p < 0.001 vs. TNF-α + NC, *n* = 5. Representative photo images and quantification of calcein-labeled THP-1 monocytes adhering to TNF-α-activated HUVECs transfected with 25 nM miR-302a-mimics **(G)** or 100 nM miR-302a-inhibitor **(H)** (*p < 0.05; **p < 0.01; ***p < 0.001 vs. TNF-α + NC; *n* = 5), scale bar, 200 μm.

To investigate the mechanism of miR-302a in regulating vascular inflammation, we examined the function of miR-302a in isolated HUVECs. As a classical cytokine for enhancing the proinflammatory activity in the endothelium, human tumor necrosis factor alpha (TNF-α) was used to induce an HUVEC inflammatory model (Jia et al., 2013; Regal-McDonald et al., 2019). As the data shown, miR-302a mimics inhibited the TNF-α-induced expression of ICAM-1 and VCAM-1 in HUVECs, whereas suppressing miR-302a increased ICAM-1 and VCAM-1 dramatically (Figures 4A,B). The secretion of ICAM-1 and VCAM-1 in HUVECs culture medium was also regulated by miR-302a consistently (Figures 4C–F). More adhesion factors will recruit more monocytes to endothelial cells. Accordingly, THP-1 cells labeled with the calcein-AM fluorescent probe were incubated with endothelial cells. Under TNF-α stimulation, the fluorescence results suggested that miR-302a inhibitor promoted TNF-α-induced adherence of monocytes to HUVECs, and overexpression of miR-302a weakened this effect (Figures 4G,H). These findings indicated that miR-302a suppressed endothelial inflammation mainly by inhibiting the secretion of adhesion factors and the adhesion of monocytes to endothelial cells.

### MiR-302a Inhibited the Nuclear Translocation of NF-κB by Modulating IKKβ-IκBα Signaling

**FIGURE 5:**
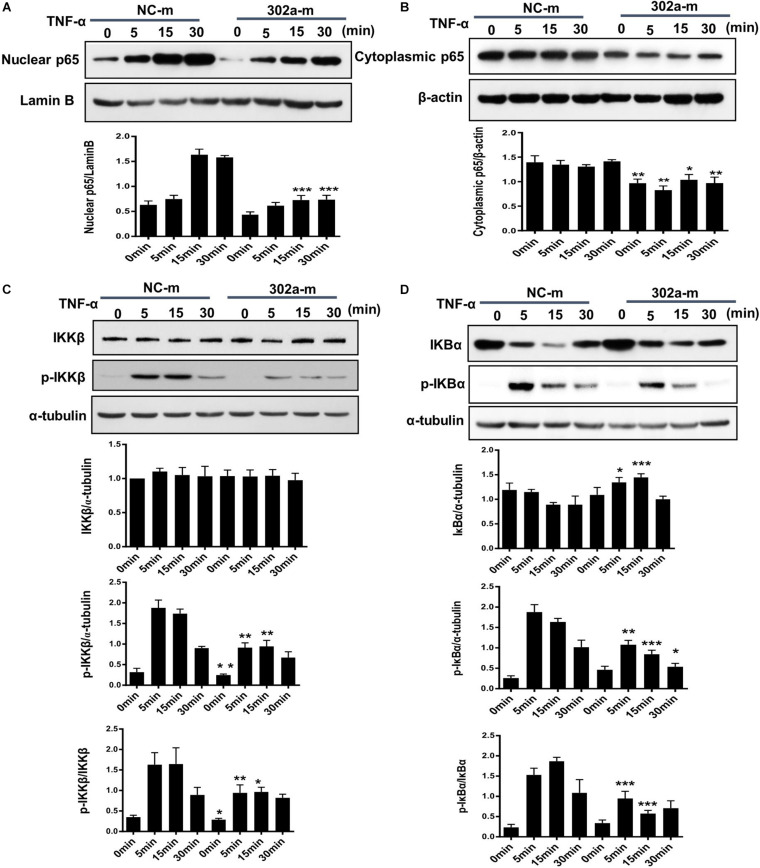
MiR-302a inhibits nuclear translocation of nuclear factor kappa-B (NF-κB) by the IκB kinase complex β (IKKβ)–inhibitors of κBα (IκBα) pathway. HUVECs were transfected with 0, 25, 50, and 100 nM miR-302a-mimics/NC-mimics for 48 h and then treated with 10 ng/ml TNF-α for 0, 5, 15, and 30 min. The expression of p65 in nuclei **(A)** and cytoplasm **(B)** were examined by western blot, *n* = 5. **(C)** The degradation and phosphorylation of IKKβ were examined by western blot, *n* = 5. **(D)** The degradation and phosphorylation of IκBα were examined by western blot, *n* = 5 (*p < 0.05; **p < 0.01; ***p < 0.001 vs. NC-m).

The NF-κB pathway is critical in the inflammation process and will be activated in TNF-α-induced HUVECs (Jia et al., 2019; Wang L. et al., 2017). Activated NF-κB induces endothelial cells to synthesize more cytokines and adhesion molecules, which recruit more leukocytes from serum to the inflamed tissue (Tang et al., 2016). To clarify whether miR-302a affected inflammation by activating the NF-κB pathway, we examined nuclear translocation of NF-κB subunit p65, which is a sign of NF-κB pathway activation. The results showed that miR-302a overexpression down-regulated the TNFα-induced translocation of p65 to the nucleus, while the expression of p65 in the cytoplasm was concomitantly down-regulated (Figures 5A,B).

Exposure to inflammatory stimuli will result in the phosphorylation of IκB kinase complex β (IKKβ), activated IKKβ regulates the phosphorylation of inhibitors of κBα (IκBα) and induces its degradation, thereby releasing NF-κB dimers, and activating the downstream NF-κB signaling pathway (Tsuchiya et al., 2017). To further illustrate how miR-302a regulates NF-κB activation, we examined the effects of miR-302a on TNFα-induced degradation and phosphorylation of IκBα and IKKβ, respectively. In endothelial cells, western blot showed that miR-302a did not affect the total protein level of IKKβ but suppressed its phosphorylation (Figure 5C), and miR-302a inhibited the TNFα-induced phosphorylation and degradation of IκBα (Figure 5D). These results demonstrated that miR-302a downregulated p65 activation by inhibiting phosphorylation of IKKβ-IκBα in endothelial cells.

### MiR-302a Modulates the IKKβ-IκBα Pathway by Targeting IRAK4

**FIGURE 6:**
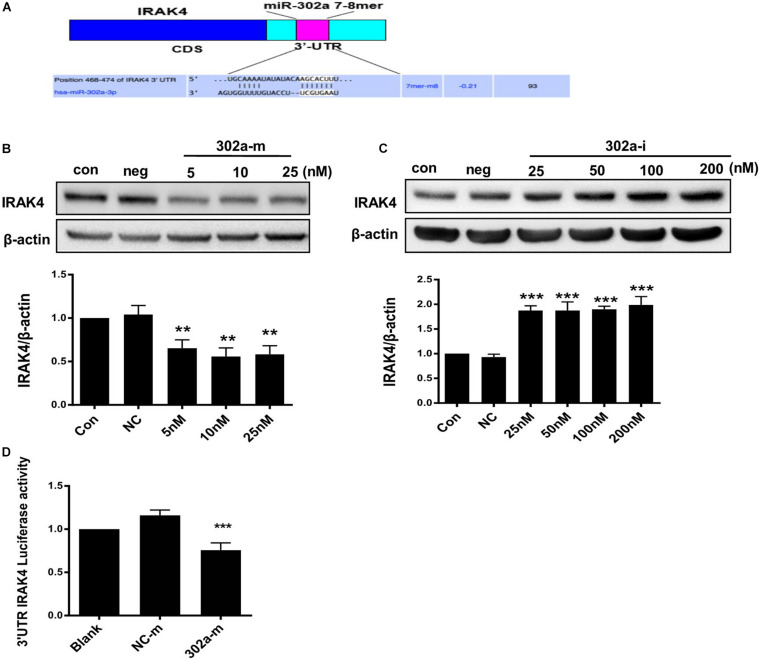
MiR-302a regulates the IKKβ–IκBα–NF-κB pathway by targeting interleukin-1 receptor-associated kinase4 (IRAK4). **(A)** Possible binding sequence of IRAK4 with miR-302a predicted by TargetScan software. The expression of IRAK4 in HUVECs transfected with miR-302a mimics (5, 10, and 25 nM) **(B)** and miR-302a inhibitor (25, 50, 100, and 200 nM) **(C)** for 48 h (**p < 0.01; ***p < 0.001 vs. Negative control; *n* = 5). **(D)** The luciferase activity of Luc-IRAK4-3’UTR in HEK 293T cells transfected with miR-302a mimics (302a-m) (***p < 0.001 vs. NC-m; *n* = 5).

As previously reported, miR-302b negatively regulates IL-1β production in monosodium urate crystal-induced inflammation by targeting IRAK4 (Ma T. et al., 2018; Zhou et al., 2014), LPS targets TLRs (Toll-like receptors) and activates IRAK4, promoting nuclear translocation of p65 by upregulating phosphorylation of IKKβ/IκBα (Wang Y. et al., 2017); thus, miR-302a may regulate IKKβ/IκBα by upstream IRAK4. We also predicted that there was a potential combining sequence between miR-302a and IRAK4 using TargetScan software (Figure 6A). To clarify the underlying mechanism, we determined the expression of IRAK4, and western blot showed that cells transfected with miR-302a mimics expressed less IRAK4 (Figures 6B,C). Furthermore, a dual-luciferase reporter experiment showed that miR-302a-mimics significantly lowered the luciferase activity of IRAK4 (Figure 6D), indicating that miR-302a inhibited IRAK4 by targeting it directly. These results suggested that miR-302a prevented NF-κB nuclear translocation by modulating the IRAK4-IKKβ-IκBα signaling pathway.

### MiR-302a Downregulated the Expression of p65 via Targeting ZFP91

**FIGURE 7:**
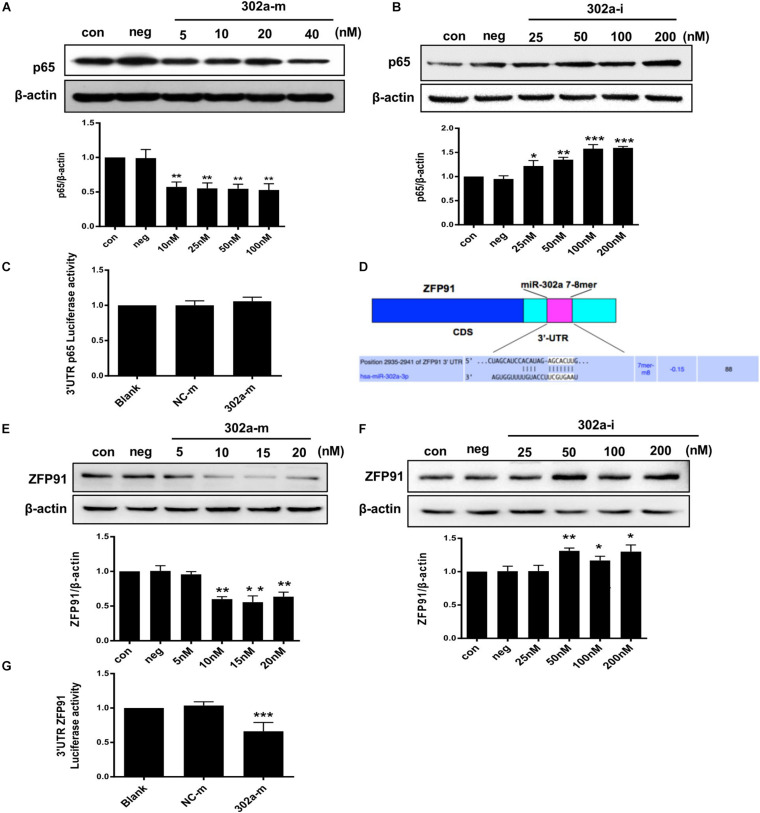
MiR-302a regulates the expression of p65 by targeting ZFP91. **(A)** P65 level in HUVECs transfected with miR-302a mimics (302a-m), **p < 0.01 vs. mimics negative control mimics (NC-m), *n* = 5. **(B)** P65 level in HUVECs transfected miR-302a inhibitor (302a-i), *p < 0.05; **p < 0.01; ***p < 0.001 vs. inhibitor negative control (NC-i), *n* = 6. **(C)** The luciferase activity of p65-3′ UTR in HEK 293T cells after transfected with miR-302a mimics, *n* = 5. **(D)** Possible binding sequence of ZFP91 with miR-302a predicted by TargetScan software. **(E)** ZFP91 expression in HUVECs transfected with miR-302a mimics, **p < 0.01 vs. NC-m, *n* = 5. **(F)** ZFP91 expression in HUVECs transfected with miR-302a inhibitor, *p < 0.05; **p < 0.01 vs. NC-i, *n* = 5. **(G)** The luciferase activity of Luc-ZFP91-3′UTR in HEK 293T cells transfected with miR-302a mimics. ***p < 0.001 vs. NC-m, *n* = 3.

Interestingly, total p65 was downregulated following miR-302a overexpression, and was upregulated when miR-302a was silenced (Figures 7A,B). These findings indicated that miR-302a does not only inhibit nuclear translocation of p65 but also downregulates its total protein.

As miRNAs regulate genes mainly by pairing with imperfect complementary sites in the 3′untranslated region (3′UTR) of target miRNAs (Bartel, 2009; Schwerk and Savan, 2015), we constructed a p65 reporter gene with 3′UTR to explore whether p65 was the direct target of miR-302a. However, the luciferase reporter assay showed that miR-302a mimics had no effect on the luciferase activity of p65-3′UTR (Figure 7C); thus, p65 was not the substrate of miR-302a and it may regulate p65 by acting on its upstream signaling. It has been reported that ZFP91 binds to the promoter sequence of NF-κB and is a transcription factor for regulating the expression of p65 (Jin et al., 2010; Paschke et al., 2016); therefore, we speculated that miR-302a may regulate p65 expression via ZFP91. We predicted that there was a possible binding sequence between miR-302a and ZFP91 using TargetScan prediction software (Figure 7D). Western blot showed that miR-302a mimics dramatically down-regulated the expression of ZFP91 in HUVEC_S_ (Figures 7E,F), suggesting that miR-302a might suppress the expression of p65 by targeting ZFP91 directly. A subsequent luciferase reporter assay showed that the luciferase activity of ZFP91-3′UTR was markedly decreased after transfection with miR302a mimics (Figure 7G), illustrating that miR-302a actually regulated the expression of p65 by binding with ZFP91 directly. These data suggested that miR-302a downregulated the expression of p65 via suppressing ZFP91 by binding with it directly.

## Discussion

**FIGURE 8:**
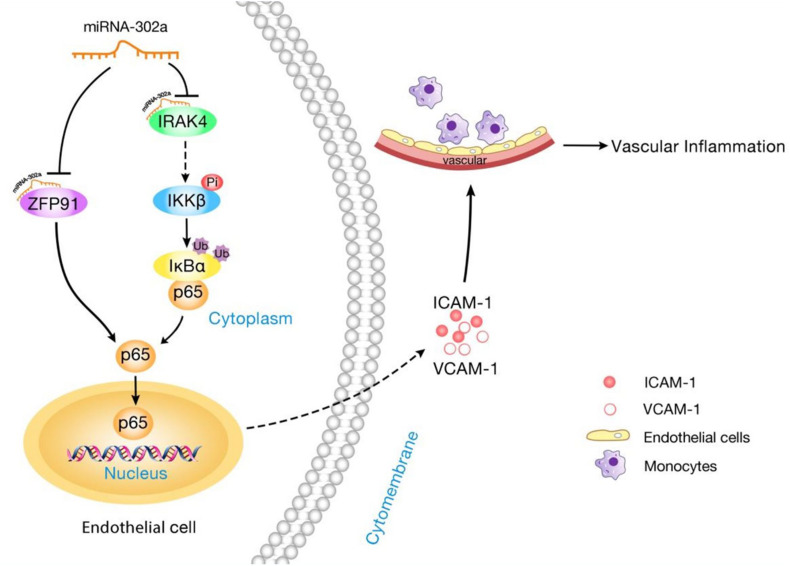
Schematic diagram of the mechanism for miR-302a in regulating endothelial inflammation. MiR-302a limits vascular inflammation by suppressing NF-κB pathway by targeting IRAK4 and ZFP91.

In this study, we showed that miR-302a is an essential suppressor of vascular inflammation. Regulation of the inflammatory response in the endothelium by miR-302a was investigated. The central findings in the present study are summarized as follows: Firstly, miR-302a was significantly decreased in mice and patients with vascular inflammation. Genetic deficiency of miR-302 aggravated LPS-induced endothelial inflammation, while overexpression of miR-302a attenuated the inflammatory response in mice. Moreover, miR-302a inhibited the secretion of inflammatory factors, resulting in reduced adhesion of macrophages to the endothelium. In the study of the underlying mechanism, we discovered that miR-302a prevented vascular inflammation by inhibiting p65 nuclear translocation through suppression of the IRAK4–IKKβ–IκBα pathway and reducing the expression of p65 by targeting ZFP91 (Figure 8).

MicroRNAs regulate approximately 30–60% of human genes for protein coding and have functions in diverse cellular processes including vascular inflammation (Dong et al., 2013). For instance, miR-24 can inhibit murine abdominal aneurysm development by suppressing aortic vascular inflammation (Maegdefessel et al., 2014). MiR-223 inhibits inflammation-related endothelial dysfunction and vascular calcification (Shi et al., 2020). Moreover, a deficiency of miR-21 in macrophages promotes plaque necrosis and vascular inflammation during atherogenesis (Canfrán-Duque et al., 2017). These findings proved the essential role of microRNAs in vascular inflammation. In the study by Liu, it was demonstrated that miR-302a exacerbates the proliferation and restenosis of vascular smooth muscle cells by increasing Akt signaling (Liu et al., 2020). However, little is known about the regulatory role of miR-302a in the endothelium. Accordingly, our *in vivo* study consistently demonstrated that knockdown of miR-302a aggravated a series of endothelial inflammatory responses, such as an increase in the secretion of inflammatory factors and upregulation of the expression of adhesion factors in the endothelium. In addition, we found that both inflammatory cell infiltration and MPO activity were also increased in the lungs and liver of miR-302 heterozygous mice, indicating that miR-302 deficiency may not only intensify endothelial inflammation in vascular tissue but also in other organs. More importantly, overexpression of miR-302a mimics effectively inhibited vascular inflammation, and weakened infiltration of inflammatory cells in the aorta, lungs, and liver. Taken together, these findings indicate the critical role of miR-302a in the development of inflammation, and show its potential for treating inflammatory vascular diseases in the future. Of course, the data here cannot exclude the possibility that the altered activity of monocytes or T cells other than endothelial cells after miR-302a treatment is also involved in regulating inflammatory response, since the *in vivo* experiments were only conducted in miR-302a constitutive knockout mice. To separate the functional role of miR-302a in endothelial cells or inflammatory cells, the tissue-specific miR-302a knockout mice are needed in the future study.

We further identified the function of miR-302a in the regulation of inflammation following *in vitro* experiments, where miR-302a mimics inhibited the expression and secretion of cell adhesion molecules such as ICAM-1 and VCAM-1 in HUVECs. In addition, overexpression of miR-302a reduced the adhesion capability of monocytes with HUVECs, while miR-302a inhibitor increased their adhesion, which is consistent with the *in vivo* histological findings.

Nuclear factor kappa-B is a nuclear transcription factor involved in a variety of pathophysiological processes and it is considered to be a classic inflammatory signaling pathway. Evidence suggests that the NF-κB pathway is pivotal for microRNAs in regulating the inflammatory reaction. MiR-199a-3p suppresses apoptosis and inflammation by regulating the IKKβ/NF-κB signaling pathway in renal tubular epithelial cells (Zhang et al., 2020). MiR-5787 attenuates inflammation by targeting TLR4/NF-κB in ischemic cerebral infarction (Zhang et al., 2019). Remarkably, miR-302c prevented the translocation of NF−κB from the cytosol to the nucleus (Gui et al., 2015). In addition, miR-302a-3p, a mature sequence of miR-302a, can inhibit receptor activator of NF-κB ligand which regulates the activation of NF-κB by binding with it (Irwandi et al., 2018). Nuclear translocation of p65 is a sign of NF-κB pathway activation. Our study findings consistently suggested that miR-302a decreased TNF-α-induced nuclear translocation of p65, but to our surprise, the total level of p65 in the cytoplasm also decreased, indicating that miR-302a not only affected the nuclear translocation of p65 but also regulated its expression. Therefore, we discussed the underlying mechanism in two ways as shown below.

Nuclear factor kappa-B binds to inhibitory protein IκB and normally exists in the cytoplasm, but under inflammation stimulation, the IκB kinase (IKK complex) is activated, phosphorylation and ubiquitination degradation of downstream IκB are induced, promoting p65 separation which is translocated to the nucleus. P65 then binds to specific sequences of downstream targets in the nucleus, activating the transcription and expression of molecules causing inflammation; thus, activation of IKKβ/IκBα is important for p65 nuclear translocation (Sandur et al., 2006; Poppe et al., 2017). Our findings demonstrated that miR-302a inhibited the phosphorylation of IKKβ without influencing its degradation, and prevented the phosphorylation and degradation of IκBα, and suppressed p65 translocation from the cytoplasm to the nucleus. It is noteworthy that miR-302b suppressed the inflammatory response to bacterial infection by targeting IRAK4, an upstream protein required for the activation and nuclear translocation of NF-κB by activating IKKβ/IκBα (Ma et al., 2018; Zhou et al., 2014). IRAK4 can be activated as TLRs are stimulated by LPS (Tomar et al., 2015). In view of the similarity between miR-302a and miR-302b, we presume that miR-302a may also target IRAK4. Consequently, the expression of IRAK4 can be markedly suppressed by miR-302a, and the luciferase reporter assay verified that IRAK4 was the direct substrate of miR-302a. Therefore, we confirmed that miR-302a inhibited the activation and nuclear translocation of NF-κB by activating IKKβ/IκBα through targeting upstream IRAK4.

Furthermore, possible ways by which miR-302a regulates the expression of p65 were explored. We studied genes which have been reported to regulate the expression of p65, and predicted which had a possible binding sequence with miR-302a using TargetScan software. We found that there was a probable binding sequence between miR-302a and ZFP91. ZFP91 can interact with NF-κB/p65 directly and is a positive regulator of p65 expression (Ma et al., 2016). Inhibition of miR-302a can downregulate ZFP91, while overexpression of miR-302a decreases ZFP91. The luciferase reporter assay further verified that ZFP91 is a binding target of miR-302a. Thus, ZFP91 is essential for miR-302a regulation of NF-κB expression. We identified two direct substrates of miR-302a. On the one hand, miR-302a could suppress the nuclear translocation of NF-κB by modulating IRAK4-IKKβ-IκBα signaling through targeting IRAK4, and on the other hand, miR-302a reduced the expression level by binding with ZFP91 directly. Interestingly, there are several reports to demonstrate that p65 and miR-302a-3p can form a feedback loop to modulate cell proliferation and migration in pancreatic ductal adenocarcinoma. MiR-302a-3p negatively regulates the expression of P65 and downstream NEAT1, meanwhile P65 and NEAT1 downregulates miR-302a-3p (Luo et al., 2019). Since p65 is increased and activated in various inflammatory conditions such as coronary artery disease, we speculated that increased p65 lowers miR-302a, the decreased miR-302a further aggravated endothelial inflammation by upregulating p65, therefore blocking p65/miR-302a-3p/p65 feedback loop may represent a novel therapeutic target to limit inflammatory diseases.

In conclusion, we demonstrated that miR-302a is an effective suppressor of vascular inflammation by regulating NF-κB signaling via suppression of ZFP91 and the IRAK4-IKKβ-IκBα pathway in endothelial cells.

## Data Availability Statement

The datasets presented in this study can be found in online repositories. The names of the repository/repositories and accession number(s) can be found in the article/Supplementary Material.

## Ethics Statement

The studies involving human participants were reviewed and approved by the Medical Research Ethics Committee of Sun Yat-sen University. The patients/participants provided their written informed consent to participate in this study. The animal study was reviewed and approved by the Medical Research Ethics Committee of Sun Yat-sen University.

## Author Contributions

J-GZ, T-TZ, and HH designed the research. J-NY, Z-LM, and YH performed the research. Q-QL and X-FL analyzed the data. T-TZ and R-PP wrote the manuscript. All authors contributed to the article and approved the submitted version.

## Conflict of Interest

The authors declare that the research was conducted in the absence of any commercial or financial relationships that could be construed as a potential conflict of interest.

## Publisher’s Note

All claims expressed in this article are solely those of the authors and do not necessarily represent those of their affiliated organizations, or those of the publisher, the editors and the reviewers. Any product that may be evaluated in this article, or claim that may be made by its manufacturer, is not guaranteed or endorsed by the publisher.

## Footnotes

1
http://www.targetscan.org
