# Supplementary figures and images for: MiR-302a Limits Vascular Inflammation by Suppressing Nuclear Factor-κ B Pathway in Endothelial Cells
Source: Front Cell Dev Biol. 2021 Aug 2;9:682574. doi: 10.3389/fcell.2021.682574 (PMC8365611; doi:10.3389/fcell.2021.682574)

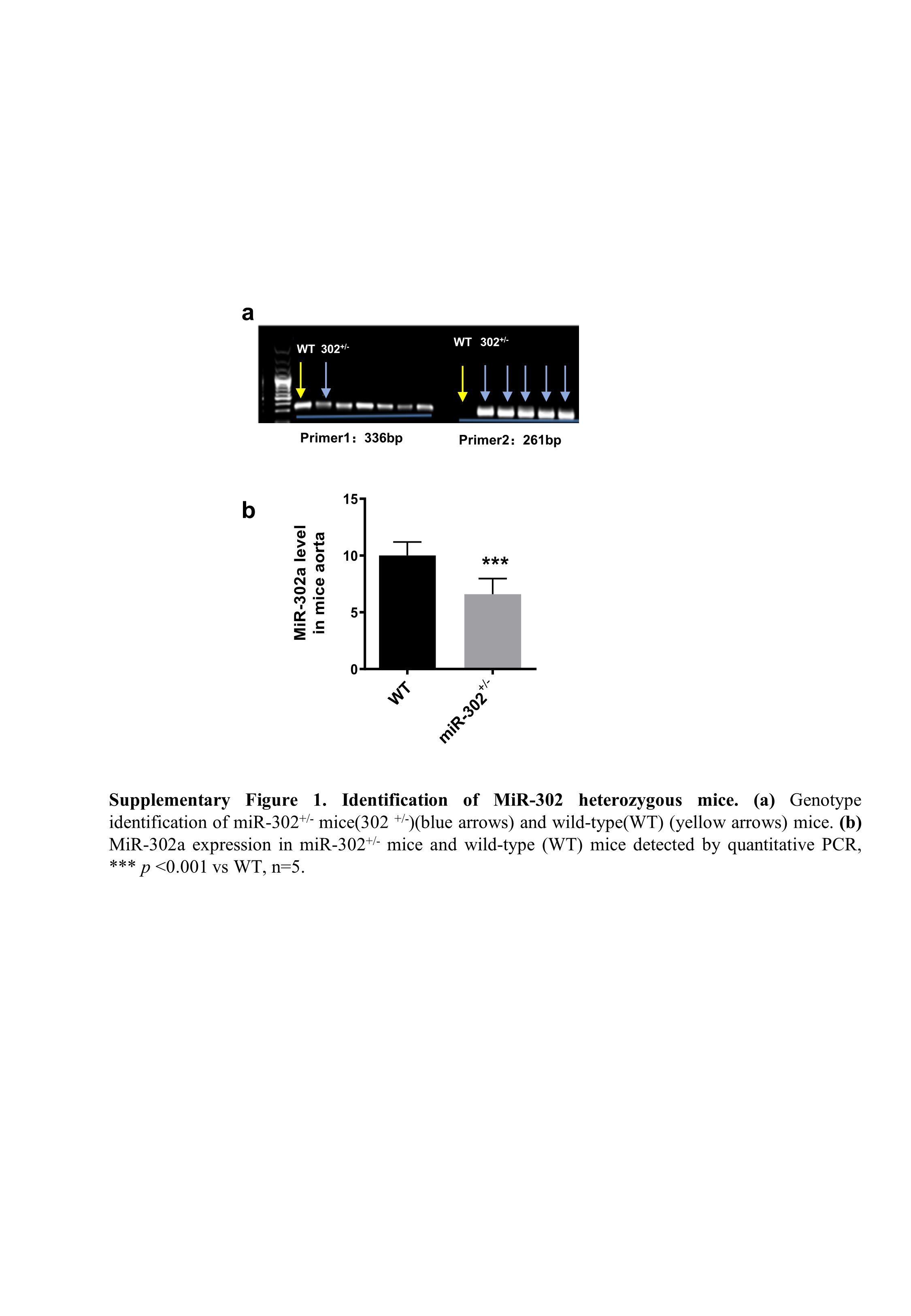

Supplement: Supplementary file 1 [file Image_1.JPEG]

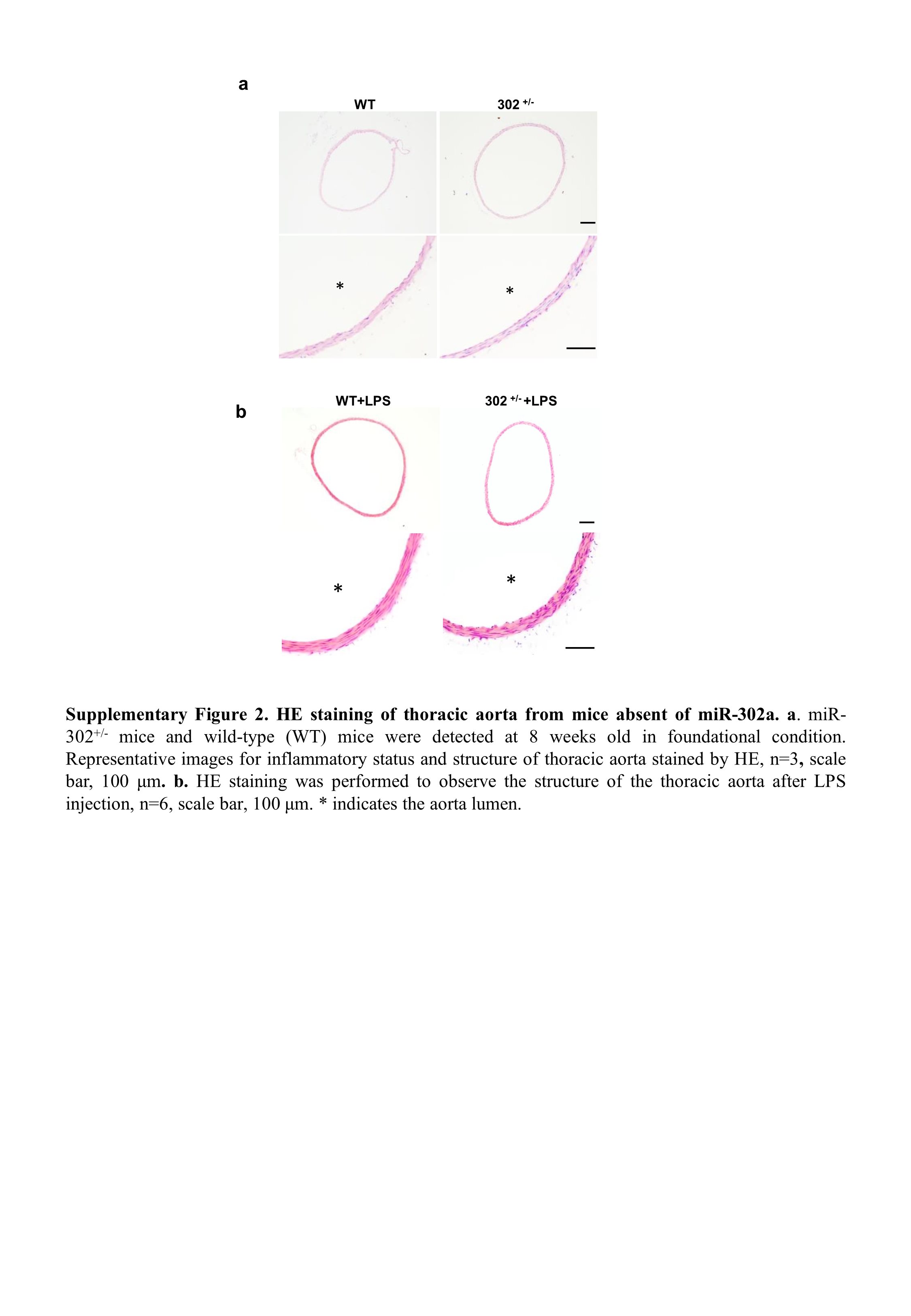

Supplement: Supplementary file 2 [file Image_2.JPEG]

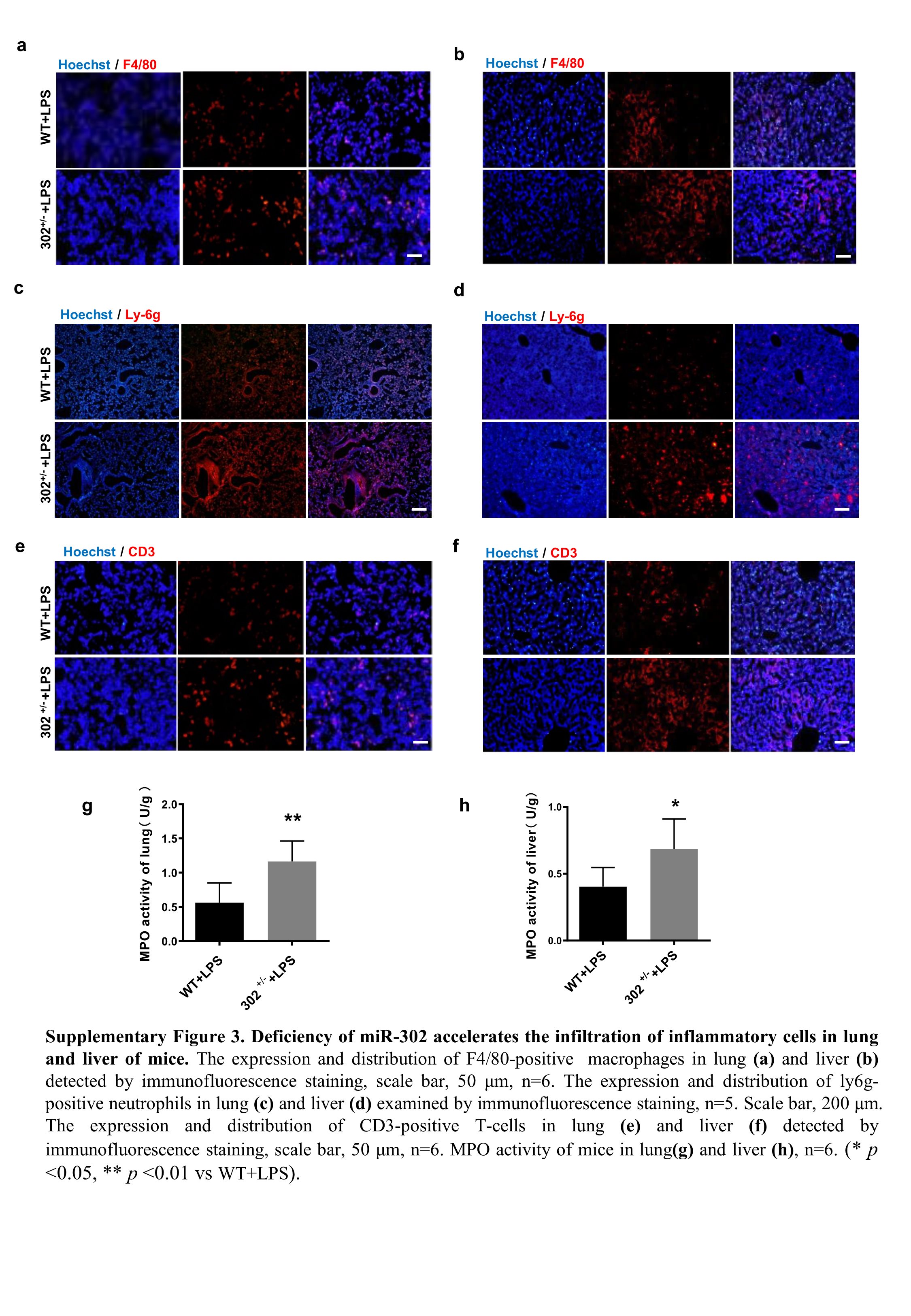

Supplement: Supplementary file 3 [file Image_3.JPEG]

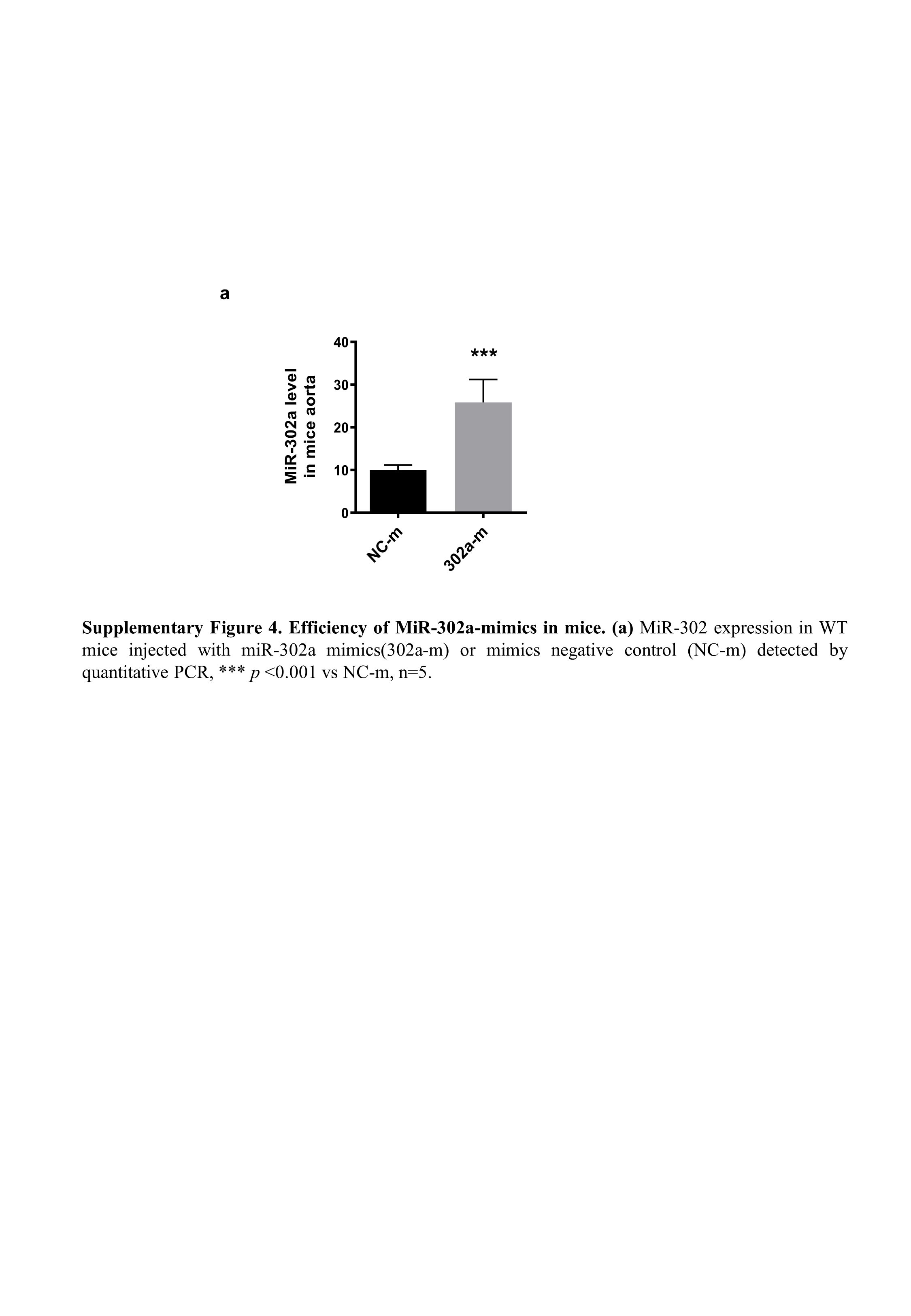

Supplement: Supplementary file 4 [file Image_4.JPEG]

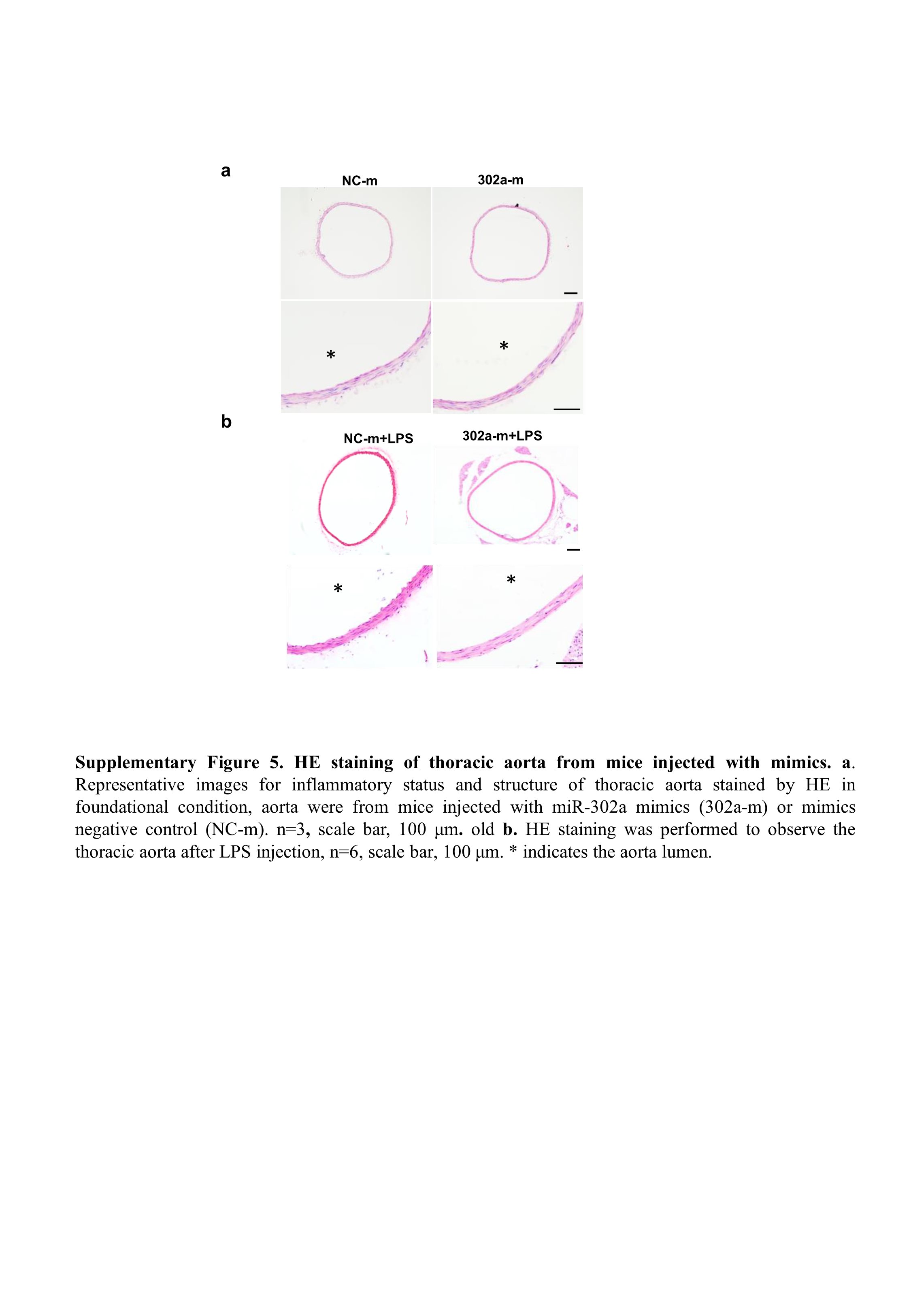

Supplement: Supplementary file 5 [file Image_5.JPEG]

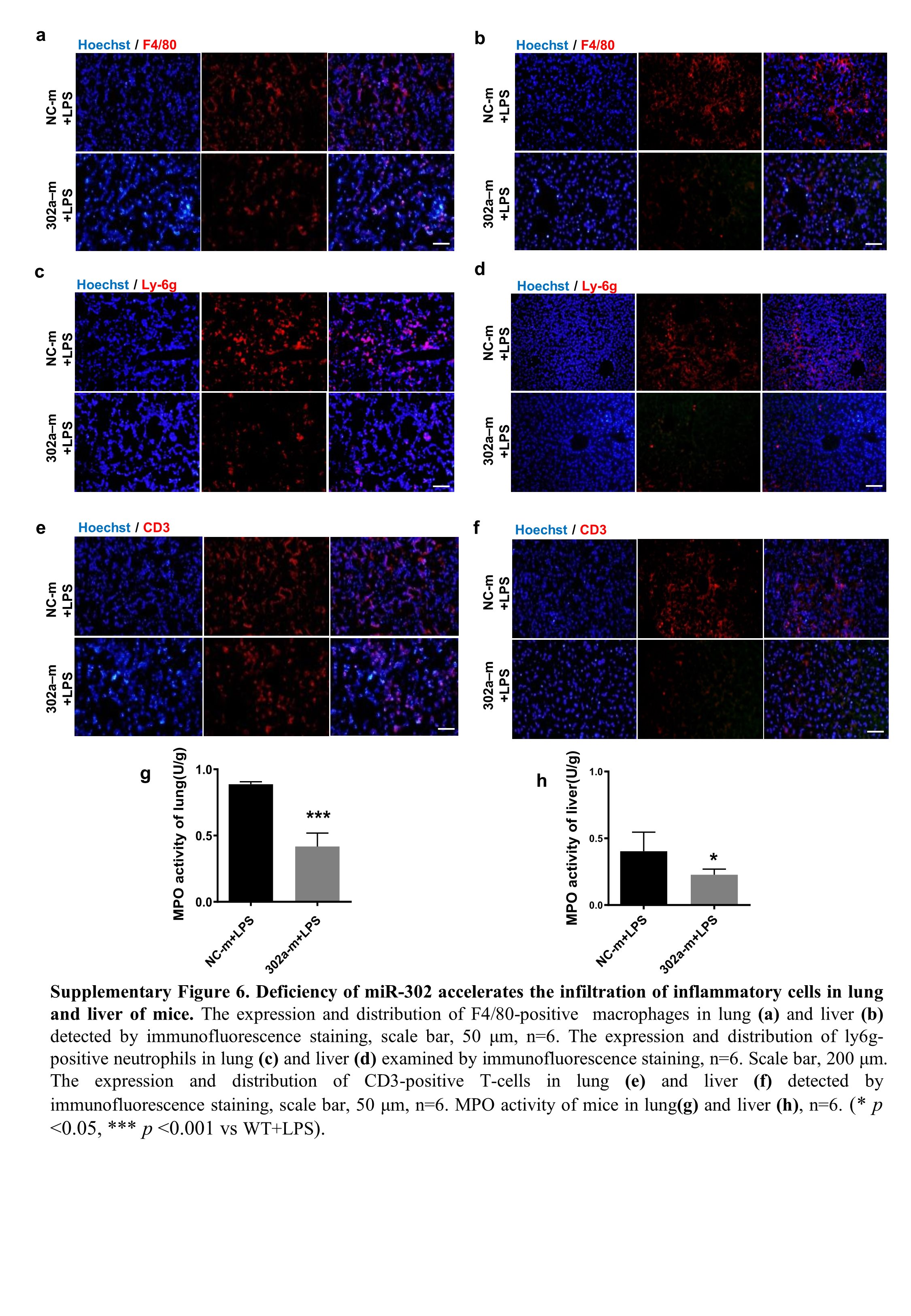

Supplement: Supplementary file 6 [file Image_6.JPEG]
